# Rapid detection of *Burkholderia pseudomallei* with a lateral flow recombinase polymerase amplification assay

**DOI:** 10.1371/journal.pone.0213416

**Published:** 2019-07-08

**Authors:** Yao Peng, Xiao Zheng, Biao Kan, Wei Li, Wen Zhang, Taozhen Jiang, Jinxing Lu, Aiping Qin

**Affiliations:** 1 Department of Pestis, National Institute for Communicable Disease Control and Prevention, Chinese Center for Disease Control and Prevention, Changping, Beijing, China; 2 Department of Diarrheal Diseases, National Institute for Communicable Disease Control and Prevention, Chinese Center for Disease Control and Prevention, Changping, Beijing, China; 3 Department of Bioinformatics, National Institute for Communicable Disease Control and Prevention, Chinese Center for Disease Control and Prevention, Changping, Beijing, China; 4 Department of Preservation Center for Standard Strain, China Institute of Veterinary Drug Control, Beijing, China; 5 Department of Hospital Antibiotics Resistance, National Institute for Communicable Disease Control and Prevention, Chinese Center for Disease Control and Prevention, Changping, Beijing, China; 6 State Key Laboratory of Infectious Diseases Prevention and Control, National Institute for Communicable Disease Control and Prevention, Chinese Center for Disease Control and Prevention, Changping, Beijing, China; University of Helsinki, FINLAND

## Abstract

Melioidosis is a severe infectious disease caused by gram-negative, facultative intracellular pathogen *Burkholderia pseudomallei* (*B*. *pseudomallei*). Although cases are increasing reported from other parts of the world, it is an illness of tropical and subtropical climates primarily found in southeast Asia and northern Australia. Because of a 40% mortality rate, this life-threatening disease poses a public health risk in endemic area. Early detection of *B*. *pseudomallei* infection is vital for prognosis of a melioidosis patient. In this study, a novel isothermal recombinase polymerase amplification combined with lateral flow dipstick (LF-RPA) assay was established for rapid detection of *B*. *pseudomallei*. A set of primer-probe targeting *orf*2 gene within the putative type III secretion system (T3SS) cluster genes was generated and parameters for the LF-RPA assay were optimized. Result can be easy visualized in 30 minutes with the limit of detection (LOD) as low as 20 femtogram (fg) (ca. 25.6 copies) of *B*. *pseudomallei* genomic DNA without a specific equipment. The assay is highly specific as no cross amplification was observed with *Burkholderia mallei*, members of the *Burkholderia cepacia*-complex and 35 non-*B*. *pseudomallei* bacteria species. Moreover, isolates from patients in Hainan (*N* = 19), Guangdong (*N* = 1), Guangxi (*N* = 3) province of China as well as in Australia (*N* = 3) and Thailand (*N* = 1) were retrospectively confirmed by the newly developed method. LODs for *B*. *pseudomallei*-spiked soil and blood samples were 2.1×10^3^ CFU/g and 4.2×10^3^ CFU/ml respectively. The sensitivity of the LF-RPA assay was comparable to TaqMan Real-Time PCR (TaqMan PCR). In addition, the LF-RPA assay exhibited a better tolerance to inhibitors in blood than TaqMan PCR. Our results showed that the LF-RPA assay is an alternative to existing PCR-based methods for detection of *B*. *pseudomallei* with a potentiality of early accurate diagnosis of melioidosis at point of care or in-field use.

## Introduction

Melioidosis, also called Whitmores disease, is an emerging infectious disease caused by the environmental bacterium *B*. *pseudomallei*. Although reported in many regions of the world, it is primarily distributed in tropical and subtropical regions. Melioidosis is estimated to account for 89,000 deaths worldwide every year [1, 2]. *B*. *pseudomallei*. has been classified as a category B bioterrorism agent by the Centers for Disease Control and Prevention of USA [3]. In China, the epidemic areas of melioidosis are mainly in Hainan, Guangdong and Guangxi province [4, 5]. Isolates from Hainan alone exhibited highly genetic diversity when tested by multilocus sequence typing (MLST) [6]. The main routes of *B*. *pseudomallei* infection are through inoculation of compromised skin, inhalation of contaminated soil during extreme weather event [7] and ingestion of contaminated water [8]. Manifestations of melioidosis are various and hard to differentiate from common pneumonia, flu or tuberculosis. In addition, *B*. *pseudomallei* is intrinsically resistance to a wide range of antibiotics, such as penicillin, ampicillin, first and second-generation of cephalosporins, gentamicin, tobramycin, streptomycin, and polymyxin [9].

Culture based method is the gold standard for diagnosing melioidosis, which typically requires 5–7 days in a highly equipped biosafety level 3 laboratory. This method has a limited diagnostic sensitivity, especially in the cases with prior antibiotic treatment. Only about one half of patients with melioidosis have positive blood cultures [10]. Therefore, it can be assumed that many cases have been under/misdiagnosed. Molecular methods such as PCR and Real-Time PCR have been prevailed for diagnosis [11]. However, they required sophisticated equipment as well as lengthy and complicated procedures. Finally, loop-mediated isothermal amplification (LAMP) assays is an alternative to PCR for rapid detection of *B*. *pseudomallei*. But the method usual consists of 4–6 primers and takes about 90 minutes to complete. In addition, the LAMP assay was insensitive to detect *B*. *pseudomallei* in clinical blood samples (only one LAMP positive out of 44 (2.3%) culture positive blood samples) [12]. A rapid and accurate diagnostic method is urgently needed.

Recombinase polymerase amplification (RPA) is a novel isothermal amplification method that can detect specific DNA or RNA with high sensitivity (less than 50 fg DNA), short turnaround time (in 5–20 minutes) and few of instrument needed. It has been utilized in detection of various pathogens including bacteria, viruses, parasites [13–17]. Basic RPA method consists of a primer pair which are able to scan and bind to the homology target DNA with the assistance of *E*. *coli Rec*A recombinase. The replication is achieved by DNA polymerase which has a strand-displacement activity necessary to extend the oligonucleotides. The displaced DNA strand was stabilized by single-strand DNA binding proteins. RPA can be conducted at wide range of temperatures from 25°C to 45°C (https://www.twistdx.co.uk/en/rpa) and even under body heat [18]. The resultant amplicon was purified then detected by agarose gel electrophoresis. Alternatively, when a third oligonucleotide probe is included, results can be analyzed by real time fluorescence with a specific instrument or by naked eyes on an oligochromotographic lateral flow strip (LF-RPA). Here we described the establishment of a TwishAmp nfo probe assay for detecting *orf*2, a gene within putative type III secretion system (T3SS) cluster genes of *B*. *pseudomallei* and verified clinical isolates of *B*. *pseudomallei* in China, Australia and Thailand. The assay is quick and easy to perform with similar sensitivity to TaqMan PCR but more tolerant to inhibitors in blood. The assay has exhibited its potential for detection of *B*. *pseudomallei* at point of need in endemic areas and emergence response in clinical settings.

## Materials and methods

### Ethics statement

Experimental protocols for handling human blood, collecting and isolating *B*. *pseudomallei* from clinical human samples were approved by the Ethical Review Committee of the National Institute for Communicable Disease Control and Prevention (ICDC), Chinese Center for Disease control and Prevention (China CDC). Written consents have been obtained from all participants prior to the study.

### Primer and probe

The primers for basic RPA and probe for LF-RPA assay were designed to target *orf2* (GenBank accession no. AF074878) following the instruction of TwistAmp DNA amplification kits (TwistDx Ltd., UK). Primer pairs were initially screened against NCBI nucleotide database using BLAST, then evaluated via basic RPA protocol (TwistDx Ltd., UK). The probe used for the LF-RPA assay was a 46 bp length of nucleotides with FAM labeled at 5´end, a tetrahydrofuran residue site (THF also referred as a dSpacer) at 30 nucleotides downstream of the 5´end and a block group (C3spacer) at 3´end. Reverse primer for LP-RPA was conjugated with biotin at 5´end. The primers for TaqMan PCR assay were selected as previously described [19]. All primers and probes were synthesized by Tianyihuiyuan. Co., Ltd. (Beijing, China) (Table 1).

**Table 1:** Sequences of primer and probe.

Assay	Name	Sequence (5´-3´) and modification	Length (bp)
Basic RPA	ORF2-1F	CGCTTCAATCTGCTCTTTCCGTTGCTGTG	28
	ORF2-1R	CTCGTTGAGGCGTGAGGTGCCCGTGTCG	27
	ORF2-2F	AGACGGCGCTTCAATCTGCTCTTTCCGTTGCTG	33
	ORF2-2R	ATCTGTTGCTAGCGGATTGTCAGGCAGTGCGTT	33
	ORF2-3F	AATCGCTCATTTCGTTCTTCCAATCATTTGTCCT	34
	ORF2-3R	GCAGGATCTTTGCTGTAGGTGAAATTCGTCGTG	33
	ORF2-4F	CGCTTCAATCTGCTCTTTCCGTTGCTGTGG	30
	ORF2-4R	CGTCATTCGCTCGATGAGGCGTGAGGTGCC	30
	ORF2-5F	CACGGCGGAGATTCTCGAATTGTCGTTGGA	30
	ORF2-5R	GCAACCACAGCAACGGAAAGAGCAGATTGAA	31
LF-RPA	LF-F	CGCTTCAATCTGCTCTTTCCGTTGCTGTG	28
	LF-R	(Biotin)-CTCGTTGAGGCGTGAGGTGCCCGTGTCG	27
	LF-P	(FAM)-GCGGCGCTGTATCGCGGCACGACGAATTTC-(dSpacer)-ACCTACAGCAAAGATCC-(C3Spacer)	46
Real-time PCR^#^	RT-F	CGTCTCTATACTGTCGAGCAATCG	24
	RT-R	CGTGCACACCGGTCAGTATC	20
	RT-P	(FAM)-CCGGAATCTGGATCACCACCACTTTCC-(BHQ1)	27

^#^ reference [19]

### Bacterial strains and genomic DNA preparation

All *B*. *pseudomallei* strains were handled in a China CDC certified Biosafety level 3 laboratory. DNA of standard *B*. *pseudomallei* strain (HN-Bp006) and culture-confirmed *B*. *pseudomallei* isolates from patients’ blood sample in Hainan (*N* = 19), Guangdong (*N* = 1) and Guangxi province (*N* = 3) during 2016–2018 were extracted using the QIAamp DNA Mini Kit (Qiagen GmbH, Hilden, Germany). The DNA concentrations were quantified by the NanoDrop ND-1000 Spectrophotometers (Callibre, USA) and stored at -20°C until use. All *B*. *pseudomallei* strains were stocked at the Department of *Yersinia* pestis, National Institute for Communicable Disease Control and Prevention, Chinese Center for Disease Control and Prevention (China CDC). DNA of *Burkholderia mallei* (CVCC-67001) was provided by Department of Preservation Center for Standard Strain, China Institute of Veterinary Drug Control. DNA of clinical strains of *Burkholderia pseudomallei* from Australia (*N* = 3, MSHR7763, MSHR7820, MSHR7995) and Thailand (*N* = 1, K96243) were gifted by Dr. Bart J. Currie, Global and Tropical Health Division, Menzies School of Health Research, Darwin, Northern Territory Australia. Members of *Burkholderia cepacia-*complex (*Burkholderia ubonensis*, *N* = 1, E105 and *Burkholderia gladioli*, *N* = 1, W38) were provided by Dr. Xuming Wang, Hainan People’s Hospital, China and Dr. Xiong Zhu, Sanya People’s Hospital, China.

### Basic RPA and by LF-RPA

The basic RPA reaction was achieved by the TwistAmp Basic kit (TwistDx, UK). A RPA reaction contained 2.4 μl forward primer (10 μM), 2.4 μl reverse primer (10 μM), 29.5 μl rehydration buffer, 12.2 μl H_2_O, 1 μl DNA template. Amplification was initialized by adding of 2.5 μl Magnesium acetate (280 mM). The mixture was incubated at indicated temperature for 5 minutes, short vortex & spin, then returned to the water bath for an additional 15 minutes. The RPA product was purified by QIAquick PCR Purification kit (QIAGEN, Hilden, Germany) and analyzed on 1.5% agarose-gel. A series of basic RPA primers were tested.

LF-RPA assay entailed 2 primers and a FAM-labeled probe (Table 1) as described by TwistAmp nfo kit (TwistDX, Cambridge, UK). A LF-RPA reaction included 2.1 μl forward primer (10 μM), 2.1 μl reverse primer (10 μM), 29.5 μl rehydration buffer, 0.6 μl probe (10 μM), 12.2 μl H_2_O, 1 μl DNA, started by adding 2.5 μl of Magnesium acetate (280 mM). The reaction was incubated at indicated temperature in a water bath for 5 minutes, short vortex & spin, then returned to the bath for an additional 15 minutes (TwistDX, Cambridge, UK). To detect amplicon by LF strip, the resultant product of RPA was diluted at 1:50 in PBS. A HybriDetect 2T dipstick was dropped in a tube containing 100 μl of the diluted product until the test line of positive control visualized (Milenia Biotec GmbH, Gießen, Germany). Result was recorded in 3 minutes.

### Optimization of temperature and time for the LF-RPA assay

To find out the optimal amplification temperature, the LF-RPA assay was carried out with 2 ng genomic DNA (gDNA) at different temperatures of 20°C, 25°C, 30°C, 37°C, 40°C, 45°C and 50°C for manufacturer’s recommended 20 minutes. Then the LF-RPA assays were performed in different reaction times to monitor the kinetic of amplification at 40°C. Reactions were incubated for 5, 10, 15, 20, 25 and 30 minutes and stopped at the time points by placing tubes on ice until further processing. The experiments were carried in single reaction and repeated twice independently.

### Evaluation of sensitivity and specificity of the LF-RPA assay

In order to assess the sensitivity of LF-RPA, gDNA of *B*. *pseudomallei* was 10-fold serial diluted. 1 μl each of diluted DNA (2 ng, 200 pg, 20 pg, 2 pg, 200 fg, 20 fg, 2 fg) was used as template. The reactions were incubated at 40°C for 20 minutes. LOD was estimated as the lowest DNA amount showing a clear test line on LF-strip. For comparison, the diluted DNA samples were tested in parallel by established TaqMan PCR protocol at 95°C for 5minutes, then 45 cycles of 95°C for 10 seconds, 60°C for 40 seconds [19].

To verify the specificity of the LF-RPA assay, gDNA (2ng) of *B*. *mallei* (*N* = 1), members of *B*. *cepacia*-complex (*N* = 2), selected strains (*N* = 5) and pools of 10 non-*B*. *pseudomallei* bacteria species (*N* = 30, DNA-MIX1 (#1–10), DNA-MIX2 (#11–20), DNA-MIX3 (#20–30),0.98–2.69 ng/strain/reaction) (Table 2) were tested for possible cross reaction. The experiment was carried out in triplicate and repeated twice.

**Table 2:** Non- *B*. *pseudomallei* bacterial strains used for specificity study.

No.	Bacteria	Strains	Concentration (ng/μl)
1	*Bacillus subtilis*	A186	18.6
2	*Bacillus sturiens*	A170	17.7
3	*Bacillus spore*	A1281	12.2
4	*Shigella Bauer*	HBSH0002	11.7
5	*Salmonella typhimurium*	JLSAL0002	12.5
6	*Acinetobacter Bauman*	301AB0145	11.7
7	*Shigella flexneri*	HBSH001	14.9
8	*Nissl spore*	A648	17.3
9	*Staphylococcus aureus*	HBSA0007	17.4
10	*Staphylococcus lugdunensis*	3012SL0005	12.9
11	*Salmonella bloom*	JLSA0003	16.2
12	*Escherichia coli*, *EIEC*	HBEC0001	21.4
13	*Bacillus sphaericus*.	A191	15.7
14	*Bacillus licheniformis*	A180	11.1
15	*Shigella Bauer*	JLSH0001	13.8
16	*Shigella Song*	HBSH0006	10.9
17	*Bacillus sphaericus*.	A184	17.0
18	*Listeria monocytogenes*	ZJLM0051	18.0
19	Drug resistant *Enterococcus*	NMVRE0001	11.3
20	*Shigella dysenter*y	HBSH0003	15.7
21	*Bacillus licheniformis thermophiles*	A197	12.1
22	*Pantoea agglomerans*	ATCC27158	9.8
23	*Bacillus cloacae*	A116	14.7
24	*Kosotobacilli sakazakh*	ATCC29544	10.9
25	*Pantoea spp*	A682	16.5
26	*Pantoea ananas*	A729	26.9
27	*Enterobacter sakazakii*	A278	12.7
28	*Enterobacter aerogenes*	CGMCC1	18.6
29	*Scattered fungi*	A895	13.0
30	*Yersinia enterocolitica*	A398	11.0
31	*Francisella tularensis*	410062	2.0
32	*Francisella philomiragia*	A740	2.0
34	*Yersinia pestis*	EV76	2.0
35	*Bacillus anthracis*	A714	2.0
36	*Burkholderia thailandensis*	W38	2.0

### Detection of *B*. *pseudomallei* in soil and blood sample by the LF-RPA

We collected soil samples (*N* = 50) from different rice plantations in Guangdong province in 2018. To isolate *B*. *pseudomallei* from the samples, 5–10 g of the soil sample were mixed in 5–10 ml of Ashdown medium with gentamycin (4 μg/ml final concentration) by vertexing, then incubated at 40°C for 2 days and spun at 1,000 rpm (939x g) for 5 minutes [20]. Supernatants were centrifugated at 6,000 rpm (3381x g) for 30 minutes then resuspended in 1ml of Ashdown medium. 100 μl/plate of them were plated on a Ashdown plate (total of 3 plates) supplemented with gentamycin [20] then incubated at 40°C for 3–7 days. The rest (ca. 600–700 μl) of the supernatants were centrifugated at 13,000 rpm (15781x g) for 5 minutes. Total DNA of the pellets were extracted by kit (Macherey-Nagel, GmßH&Co.KG, Germany) and DNA concentration was measured (NanoDrop ND-1000). 1μl of the DNA (range from 3.7 to 25.6 ng) was used for screening *B*. *pseudomallei* by TaqMan PCR. Selected samples were further verified by the LF-RPA assay.

To determine the diagnostic potential of the LF-RPA assay, we tested the assay on *B*. *pseudomallei*-spiked defibrinated rabbit blood (Lefeikangtai, Beijing, China), soil or LB medium. Before spiking, *B*. *pseudomallei* colony forming units (CFU) was determined by plate-counting technique. Briefly, OD_600_ = 1 of *B*. *pseudomallei* culture was 10-fold serial diluted (10^−1^–10^−8^), 20 μl aliquots of appropriate dilutions (10^−7^, 10^−8^, 10^−9^) were dripped onto LB plate in triplicate and incubated at 40°C for 48 hours before CFU counting. 100 μl of each serial dilution containing 4.2x10^5^ CFU, 4.2x10^4^ CFU, 4.2x10^3^ CFU, 4.2x10^2^ CFU, 4.2x10^1^ CFU and 4.2 CFU of *B*. *pseudomallei* respectively were inoculated in 900 μl of blood. The mixtures were centrifuged at 13,000 rpm (15781x g) for 5 minutes, total DNA of pellets were extracted with Qiagen Blood &Tissue kit. 1μl of DNA was tested by the LF-RPA and TaqMan PCR. Under this protocol, LOD on spiked blood or LB were expressed as the lowest CFU/ml at which concentration the LF-RPA generates a visible test line on strip.

In order to estimate the LOD of the mocked soil samples, 100 μl of each serial dilution (10^−3^–10^−8^) containing 4.2x10^5^ CFU, 4.2x10^4^ CFU, 4.2x10^3^ CFU, 4.2x10^2^ CFU 4.2x10^1^ CFU and 4.2 CFU of *B*. *pseudomallei* were mixed well with 2 g of soil sample in 900 μl of PBS, spun at 1,000 rpm (939x g) for 5 minutes. Supernatants were centrifugated at 13,000 rpm (15781x g) for 5 minutes. Total DNA of the pellets were extracted then 1μl of DNA was analyzed by the LF-RPA or TaqMan PCR. Under this protocol, LOD was expressed as the lowest CFU/g generating a visible test line on strip.

### Inhibition of the LF-RPA by blood

To explore the possibility of inhibitory effect of blood on the LF-RPA and TaqMan PCR, defibrinated rabbit blood or horse blood (Lefeikangtai, Beijing, China) were proportionally added into the reaction directly at final ratio of 0%, 1%, 5%, 10%, 12% and 15% (v/v). Standard LF-RPA and TaqMan PCR reactions were performed as described above.

To mimic real clinical sample, defibrinated normal human blood (Lefeikangtai, Beijing, China) was spiked with *B*. *pseudomallei* at final concentration of 5000 ± 545 CFU / ml, 500 ± 54 CFU / ml, 50 ± 5.45 CFU / ml, 5 ± 0.545 CFU / ml, 0.5 ± 0.0545 CFU / ml, 0 CFU / ml then boiled for 5 minutes, centrifugated at 13,000 rpm (15781x g) for 5 minutes. 1μl supernatant of each sample was analyzed by the assay. DNA of *B*. *pseudomallei* was serial diluted by normal human blood at final concentration of 2.5 ng/ml, 0.25 ng/ml, 25 pg/ml, 2.5 pg/ml and 0 pg/ml. 1μl of each mock sample was run by the LF-RPA. LODs were defined as the lowest CFU/ml of *B*. *pseudomallei* or fg of gDNA in mock sample that generates a positive test line. The experiment was repeated two times.

## Results

### Design and screening of primers

**Fig 1:**
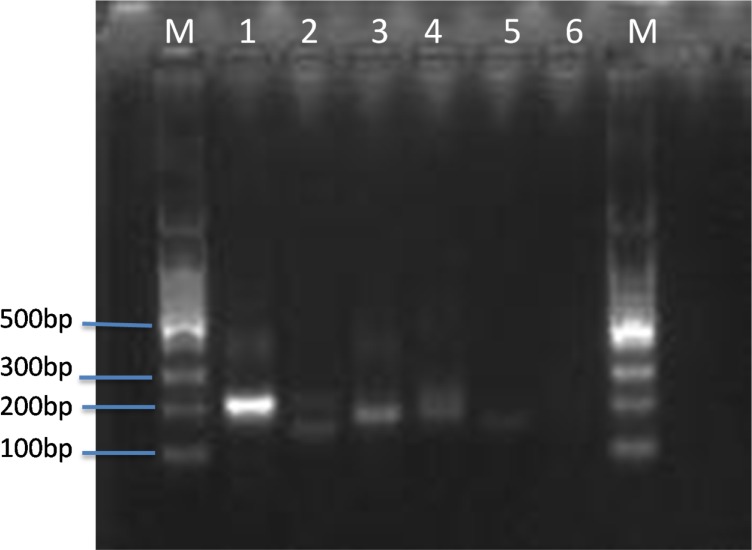
Primers screening for basic RPA. Several sets of primers targeting *orf2* gene of *B*. *pseudomallei* were tested for amplification efficiency by basic RPA (2 ng gDNA as template, at 40°C for 20 minutes). Lane 1–5 are primers of 1F/1R, 2F/2R, 3F/3R, 4F/4R, 5F/5R. lane 6 no primers control. M DNA ladder.

All primer candidates within the *orf*2 gene (Table 1) were designed following the manufacturer’s instructions and pair screened by basic RPA kit (TwistDx Ltd., UK). In contrast to normal PCR, RPA primers require longer oligonucleotides, typically 30–35 base pair (bp) in order to stimulate homology recombination with the assistance of recombinase. Primers were paired and screened in a single tube reaction without the addition of the probe. Reaction conditions such as temperature, incubation time and primer concentration were optimized. As shown in Fig 1, ORF-1F/ORF-1R, a 198-bp amplicon, exhibited the best efficiency (lane1) at 40°C for 20 minutes and therefore, was chosen in the follow-up experiments.

### Optimization of reaction temperature and time

**Fig 2:**
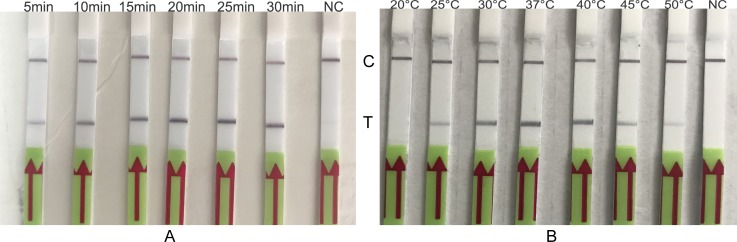
**Optimization of temperature(A) and time(B) for the LF-RPA.** gDNA of *B*. *pseudomallei* (2 ng) was used in each reaction at indicated temperatures for 20 minutes (panel A) or at 40°C for indicated time (panel B). NC negative control. C, control line; T, test line. These experiments were repeated three times.

To evaluate the optimal amplification temperature, LF-RPA assay was performed at indicated temperatures for 20 minutes as recommended by manufacture (Fig 2A). The band density of test line on the strips varies with temperature over a wide range. Decent amplification was able to achieve at 30°C-40°C. Therefore, the assay is suitable to be performed under human body temperature. This is of an advantage for operating the assay with low resource settings. The whole assay takes 30 minutes or less.

In order to monitor the kinetic of the LF-RPA amplification as well as to estimate the optimal reaction time, the assay was performed at 40°C for different incubation time. As the results show in Fig 2B, test line could be observed in as few as 5 minutes. The test line became intensified as time extended to 10 minutes or longer. Taking the detection efficiency and sensitivity into account, 20 minutes were selected for the LF-RPA assay.

### Evaluation of the sensitivity and specificity of the LF-RPA assay

**Fig 3:**
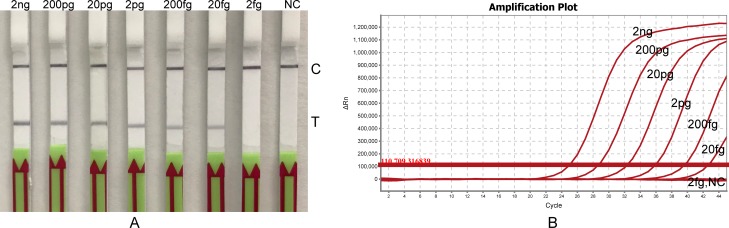
**Sensitivity of the LF-RPA(A) and Real-Time PCR(B).** Serial diluted gDNA of *B*. *pseudomallei* (from 2 ng to 2 fg/reaction) was tested by LF-RPA at 40°C for 20 minutes (panel A) and by Real-Time PCR at 95°C for 5minutes, then 45 cycles of 95°C for 10 seconds, 60°C for 40 seconds (panel B). NC negative control C, control line; T, test line. The experiments were repeated twice with the same result.

To estimate the LOD of *B*. *pseudomallei*, gDNA from 2 ng to 2 fg were tested by the LF-RPA assay. The results shown in Fig 3A that the current method allows to detect as low as 20 fg of gDNA. (or 25.6 copies, when amount of DNA was converted to copy number by the following formula: number of copies = (amount of DNA (ng) x 6.022 x 10^23^) / (length of DNA (bp) x 10^9^ x 650)). Therefore, the sensitivity of LF-RPA is as good as the TaqMan PCR (Fig 3B). LOD of the assay on CFU of *B*. *pseudomallei* is 420 CFU/ml when *B*. *pseudomallei* is in LB medium (see below).

**Fig 4:**
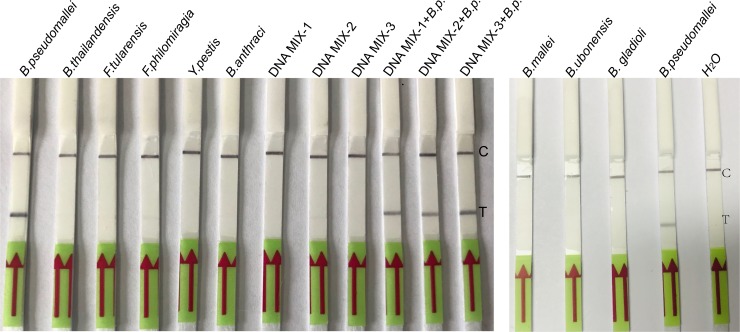
Specificity of the LF-RPA assay. LF-RPA reactions (at 40°C for 20 minutes.) were conducted with gDNA (2 ng/reaction) of indicated bacteria (lane 1–6 and lane 13–15) or pools gDNA of non-*B*. *pseudomallei* strains (0.98–2.69ng/each strain/reaction, lane 7, 8, 9). Lane 10, 11, 12 non-*B*. *pseudomallei* strains gDNA pools spiked with 2 ng/reaction of HN-Bp006 gDNA respectively. C, control line; T, test line. This experiment was repeated three times with the same result.

To investigate the specificity of the LF-RPA, gDNA of a panel of bacterial pathogens were extracted individually and 2 ng were employed for the assay. Positive result could be generated only with *B*. *pseudomallei* strain, but not with the following bacteria: *B*. *mallei*, *B*. *ubonensis*, *B*. *gladioli*, *B*. *thailandensis*, *Francisella tularensis*, *Francisella philomiragia*, *Yersinia pestis*, *Bacillus anthracis*. Pools of DNA from 10 non-*B*. *pseudomallei* bacteria species at final concentration of 0.98–2.69 ng/each strain/each reaction (Table 2) were all negative (Fig 4). No cross-reaction was observed with other members of the *Burkholderia* family and selected non-*Burkholderia* bacteria species, implying a high analytic specificity of this assay. Similar experiments were performed twice with the same results. Hence, the LF-RPA assay exhibited a trustworthy specificity.

### Validation of clinical isolates and evaluation of *B*. *pseudomallei*-spiked blood and soil samples

**Fig 5:**
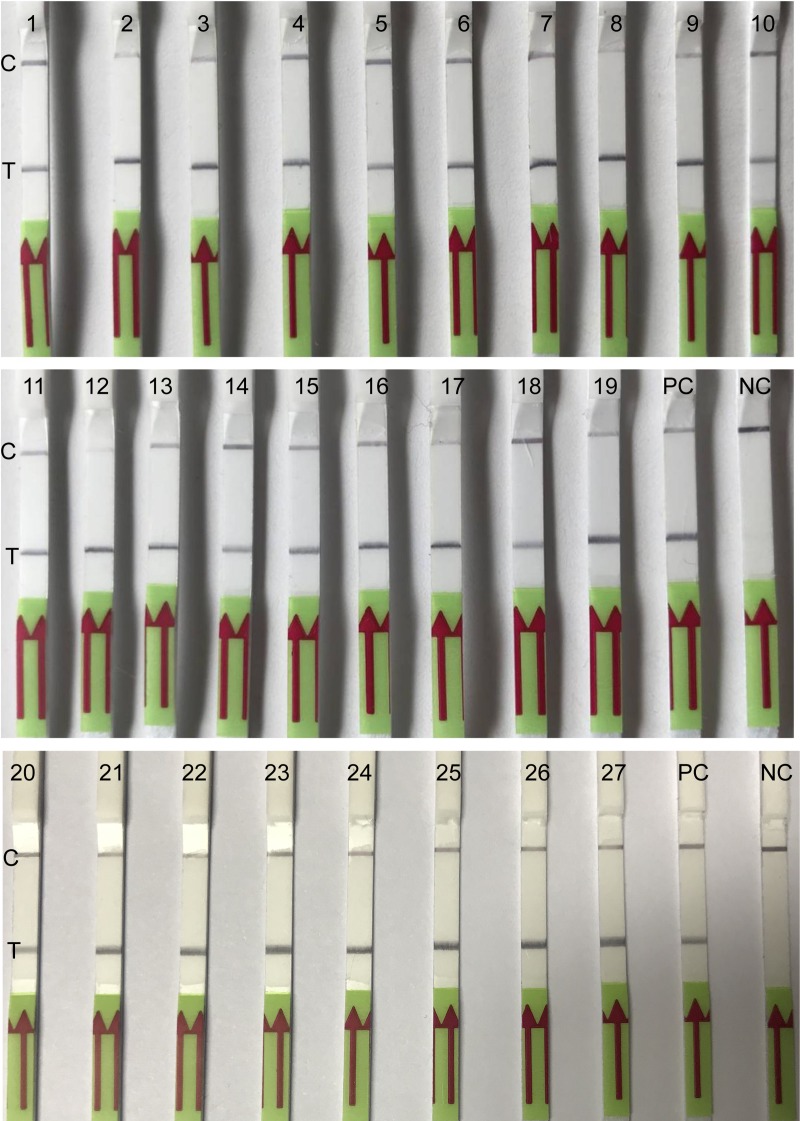
Analysis of clinical isolates by the LF-RPA assay. gDNA (2 ng/reaction) of *B*. *pseudomallei* strains, collected from melioidosis patients in China, Australia and Thailand were retrospectively confirmed by the LF-RPA. lane 1–19 from Hainan province, lane 20 from Guangdong province, lane 21–23 from Guangxi province, lane 24–26 from Australia, lane 27 from Thailand. PC positive control (2 ng of HN-Bp006 gDNA), NC negative control (water). C, control line; T, test line.

*B*. *pseudomallei* isolates collected from clinical patients of Hainan (*N* = 19), Guangdong (*N* = 1), Guangxi province (*N* = 3) during 2016–2018, and *B*. *pseudomallei g*DNA from Australia (*N* = 3), Thailand (*N* = 1) were formerly culture-confirmed. Here we further verified these strains by the LF-RPA assay. 100% of the isolates (27/27) demonstrated a clearly visible line as positive control (HN-Bp006 gDNA) indicating that the assay is a reliable method to detect *B*. *pseudomallei* (Fig 5). This experiment was conducted two times with same result.

**Fig 6:**
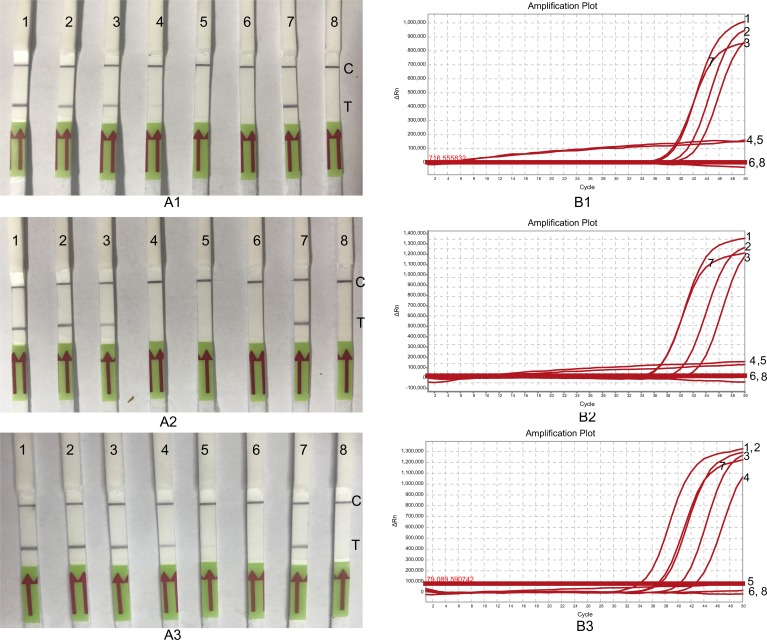
LODs of the LF-RPA and Real-Time PCR for spiked samples. *B*. *pseudomallei* was 10-fold serial diluted and CFU/ml was estimated by plate-counting method. Each of dilution was inoculated in blood (A), soil (B) or LB medium (C) respectively. Total DNA was extracted and tested by the LF-RPA (A1, B1, C1) and Real-Time PCR (A2, B2, C2). Lane 1, 4.2×10^5^, lane 2, 4.2×10^4^, lane 3, 4.2×10^3^, lane 4, 4.2×10^2^, lane 5 4.2×10^1^, lane 6, 4.2. Lane 7, 2ng of HN-Bp006 gDNA, lane 8, water control. C, control line, T, test line.

*B*. *pseudomallei* is a soil dwelling pathogen. In order to evaluate the feasibility of the LF-RPA assay as a surveillance tool, 50 soil samples were collected from Guangdong province in 2018. Total DNA was prepared with soil DNA isolation kit as described in materials and methods. The TaqMan PCR and the LF-RPA assay were conducted to detect *B*. *pseudomallei*. All the samples (*N* = 50) were TaqMan PCR negative (S1 Fig). As expected, no *B*. *pseudomallei* was isolated from these samples by culture method. In this case, we spiked the soil samples with HN-Bp006 bacterial cells at different CFU/g to evaluate the capability of this assay to detect *B*. *pseudomallei* in soil sample. LOD of the LF-RPA and TaqMan PCR for spiked soil were both estimated at 2100 CFU/g (Fig 6).

*B*. *pseudomallei* is commonly isolated from blood of melioidosis cases. To explore the possibility of the LF-RPA as a potential diagnostic mean at point of care, *B*. *pseudomallei* was spiked in rabbit blood. The mocked clinical samples were tested by LF-RPA and TaqMan PCR (Fig 6). The LOD for both TaqMan PCR and the LF-RPA assay were 4.2×10^3^ CFU/ml (Table 3). We concluded that the capability of the LF-RPA assay to detect *B*. *pseudomallei* from mocked field samples is as effective as TaqMan PCR. However, as expected, LODs of the LF-RPA on the *B*. *pseudomallei*-spiked blood (4200 CFU/ml) and soil (2100 CFU/ml) samples are higher than that bacteria in the LB culture medium (420 CFU/ml) (Table 3), suggesting that field samples such as blood or soil, may contain inhibitory substance (s) interfering both of LF-RPA and TaqMan PCR assay.

**Table 3:** LODs of the LF-RPA and Real-Time PCR.

Assay	LOD in		
	LB (CFU/ml)	Blood^!^(CFU/ml)	Soil (CFU/g)
Real-Time PCR	420	4200	2100
LF-RPA	420	4200	2100

^!^rabbit or horse blood

### Effect of blood on the LF-RPA assay

**Fig 7:**
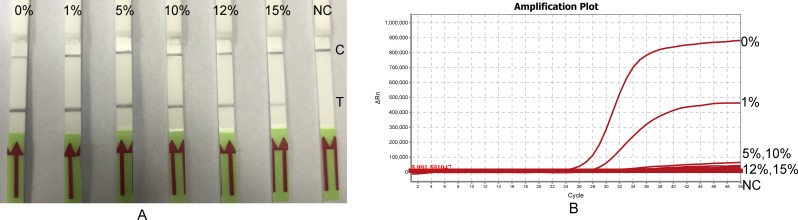
Effect of blood on the LF-RPA assay and Real-Time PCR. Defibrinated rabbit blood was included in reactions of the LF-RPA (panel A) or Real-Time PCR (panel B) at the indicated percentages (v/v). The LF-RPA was able to detect 1 ng gDNA of *B*. *pseudomallei* at the presence of 12% of blood. Instead Real-Time PCR lost detectable signal when blood concentration in reaction was more than 1%. NC negative control. C, control line, T, test line.

The enzymatic nucleic acid amplification can be affected by numerous substances. To study the interference of blood on the LF-RPA assay, standard LF-RPA reaction (2 ng template DNA, final volume of 50 μl) was modified with rabbit or horse blood at ratio of 0%, 1%, 5%, 10%, 12% and 15% (v/v). Amplifications of the LF-RPA were achieved when the percentage of blood in reaction volume was less than 12%. In contrast, TaqMan PCR lost detectable signal when exceeded 5% (Fig 7). Increasing DNA template in the assays (from100 pg to 1 ng) counteracts this inhibition in some extent. Same inhibitory pattern was observed when horse blood was tested, suggesting that this inhibition is not blood type dependent (Table 4). Moreover, when the ratio of blood in a standard PCR or TaqMan PCR reaction was more than 1%, floccules formed during the denature step of the assays. This was not the case of LF-RPA assay (S2 Fig). In all, the LF-RPA assay tolerates the inhibitors presented in blood better than TaqMan PCR.

**Table 4:** Inhibition of blood on TaqMan PCR and the LF-RPA assay.

Assays	DNA	Blood^!^ ratio in reaction (%)					
		0	1	5	10	12	15
Real-Time PCR	100 pg	+*	-	-	-	-	-
	1 ng	+	+	-	-	-	-
	10 ng	+	+	-	-	-	-
LF-RPA	100 pg	+^※^	+	+	+	-	-
	1 ng	+	+	+	+	+	-
	10 ng	+	+	+	+	+	-

* Ct< 35 as positive

※ see visible Test-line by naked eyes on strip

^!^ rabbit or horse blood

+ positive

- negative.

In order to estimate the LOD on real clinical blood sample, normal human blood (NHB) was spiked with bacterial cells (CFU) or gDNA of *B*. *pseudomallei*. LOD on blood (5000±544 CFU/ml) was significantly higher (p<0.001, Student’s t-Test) than on PBS control (5±0.45 CFU/ml), suggesting the inhibitory effect of blood to the LF-RPA. But the LOD of gDNA-spiked blood (25±0 fg) remains the same level as gDNA-spiked PBS (**Table 5**). Since sample boiling step was skipped for the gDNA spiked NHB, it is tempting to speculate that the inhibitor (s) in blood most likely comes from blood cells.

**Table 5:** LOD of the LF-RPA on spiked normal human blood.

Spiked	LOD in	
	PBS	NHB^$^
CFU (CFU / ml)^**&**^	5±0.45	5000±544
gDNA (fg) ^@^	25±0	25±0

^**&**^ CFU spiked samples were boiled for 5 minutes before being used as template.

^@^1μl of gDNA spiked samples were used directly as templates.

^**$**^ normal human blood.

## Discussion

First introduced in 2006, RPA represents an innovative DNA Isothermal detecting technology that has been used to detect a range of pathogens. It is an alternative to existing PCR-based method. One of the advantages of RPA method is its minimum equipment requirement. The assay was conducted successfully under human body heat [18]. The LF-RPA assay reported here to detect *B*. *pseudomallei* is as sensitive as TaqMan PCR and is able to amplify DNA to detectable levels in a temperature range of 25°C to 50°C, indicating it is feasible to carry the assay under human body temperature. This is of a great advantage for field application in low resource settings. The assay possesses a comparative LOD with LAMP assay which detects BPSS1406, a gene also located within the cluster genes encoding T3SS of *B*. *pseudomallei* [12]. The *orf2* gene was successfully used to detect *B*. *pseudomallei* of 27 clinical isolates but was negative with 35 non-*B*. *pseudomallei* bacteria species and *B*. *mallei*, a relative of *B*. *pseudomallei* and also a category A bioterrorism relevant organism. In addition, the assay can distinguish *B*. *pseudomallei* from members of *B*. *cepacian-*complex and non-human pathogen of *B*. *thailandensis* [21], suggesting the high specificity of this gene for detecting of *B*. *pseudomallei*. The *orf*2 gene, within the type III secretion system gene cluster of the *B*. *pseudomallei* (GenBank accession no. AF074878), was no significant similarity to other *Burkholderia* subspecies, and previous targeted to distinguish *B*. *pseudomallei* from other microbial species by TaqMan PCR [18, 22]. The LF-RPA method is a very rapid technique, providing instructive information in less than 30 minutes (20 minutes reaction and 5 minutes detection) with a high sensitivity (LOD of 20 fg (ca.25.6 copies) on pure gDNA of *B*. *pseudomallei*, or 5±0.45 CFU/ml on *B*. *pseudomallei* cells. LOD of LF-RPA for detection of *Salmonella* was 10.5 CFU/ml [23]. It seems that template prepared by boiling sample at 95°C for 5 minutes is better than by isolating gDNA with a purification kit. As demonstrated in this study, the LOD of boiling method is 5 CFU/ml verse 420 CFU/ml of kit purified gDNA. This may due to the loss of gDNA during the purification procedures.

The absence of typically clinical symptom of melioidosis renders early diagnosis difficult. Serology assays such as Indirect Hemagglutination Assay (IHA) remain the most commonly used method for melioidosis diagnosis. Polysaccharide-based latex agglutination assays have been established and evaluated. However, 38% of healthy adults were IHA seropositive in endemic areas and 13% of patients were IHA negative despite culture positive of *B*. *pseudomallei* [24]. Thus, the agglutination assays appear not to be an ideal serologic method for diagnosing melioidosis [25]. Isolation of *B*. *pseudomallei* from patient is the conclusive diagnosis of melioidosis. It usually takes 5–7 days, in which time-frame patient may have been treated ineffectively and results in a higher fatality [26]. Immunofluorescence microscopy, PCR and TaqMan PCR methods were utilized to diagnose clinical patient and provide helpful evidence. However, they need expensive instruments and well-trained technician that are not adapted to resource limited areas. RPA possesses an advantage over current molecular diagnosis methods, it may be able to detect clinical sample which had been missed by PCR [27]. Hemoglobin, lactoferrin and immunoglobulin G in blood for example are potential PCR inhibitors [28]. Residues of detergents, salts and ethanol carried over during DNA isolation may hinder PCR reaction [29]. Although inhibitory effects of whole blood were reported [30], RPA had exhibited a certain tolerance to crude samples or crude materials with minimal processing [31]. The strategy reported here will improve diagnostic reliability and enable to make early accurate diagnosis of melioidosis.

Frequent specimens for *B*. *pseudomallei* include soil from environment and blood from clinical patient. The LF-RPA performed well with *B*. *pseudomallei* spiked soil and blood samples at a LOD of 2100 CFU/g and 4200 CFU/ml respectively, higher than the LODs of *Salmonella*-spiked chicken breast (105 CFU/g) or milk (105 CFU/ml) [23]. This discrepancy probably stems from the different method of template preparation and inhibitory substance in different kind of samples. In this study, only mock samples were tested. Future studies will aim to assess the assay with real clinical specimens, such as blood, pus, urine and body fluids. Even though, the satisfactory results of successful detection *B*. *pseudomallei* from simulated samples make us speculate that it is a promising method for clinical use.

A constraint of the LF-RPA for routine field application is the time-consuming DNA extraction steps involved in this study. However, not like TaqMan PCR, the LF-RPA demonstrated a better tolerance to the inhibitors present in blood and other common PCR inhibitors [32]. We observed that when blood ratio was more than 1% (v/v) in a PCR or TaqMan PCR reaction, visible flocculent precipitation formed during the 95°C denature step. Also, the color of blood in reaction may interfere the result analysis of TaqMan PCR and LAMP. On the other hand, LF-RPA is conducted at a constant lower temperature (40°C in this study) bypassing this obstacle. Moreover, no alteration of sensitivity was observed when the percentage of blood was less than 10% in the LF-RPA reaction. Template in our standard LF-RPA was 1μl out of 50 μl reaction volume (2%). RPA exhibited a relatively tolerance to the present of 10% blood in reaction, therefore, blood sample is feasible to be applied directly as template. This notion is further supported by the assay results of *B*. *pseudomallei*-spiked NHB.

Risk of false positive exists for isothermal amplification methods [33]. We observed false positive in the no-template control (water). This assay noise could be abrogated when the amplification product was diluted at a ratio of 1 to 100 instead of recommended 1 to 50 before conducting the lateral flow strip step [15]. Dimer formed between probe and 3´primer may play a role in the false positive [34]. We found that separate mixing of RPA reagents and template areas greatly reduce this risk. Lack of internal control impedes the practical application of this novel technique. This issue can be overcome by spiking external control during template preparation [35] and developing of a duplex LF-RPA assay [36].

In summary, we first applied the isothermal recombinase polymerase amplification with later flow strip (LF-RPA) assay for detection of *B*. *pseudomallei*. Although further studies are required to fully evaluated its practicability, it is a promising tool with advantages over currently available DNA diagnostic systems for melioidosis diagnosis.

## Supporting information

S1 Fig Screening for *B*. *pseudomallei* from soil samples collected from Guangdong province. Soil samples (*N* = 50) were collected from rice paddies in Guangdong, a province next to Hainan. Total DNA of each sample (5–10 grams) was extracted and screened for *B*. *pseudomallei* by TaqMan PCR (upper panel). Selected 10 samples which ΔRn value elevated at Ct 20 in TaqMan PCR assay were tested by the LF-RPA assay (lower panel). PC positive control (2 ng of HN Bp-006 gDNA), NC negative control (water). C, control line; T, test line.
(EPS)

S2 Fig Formation of flocculent precipitation during PCR and TaqMan PCR, but not the LF-RPA. Defibrinated rabbit blood were proportionally added in the reactions of the LF-RPA, standard PCR or TaqMan PCR at the final concentration of 0%, 1%, 2% 3%, 4%, 5%,10% and 15% (v/v). The LF-RPA was conducted at 40°C for 20 minutes. PCR was performed at 95°C for 5minutes, then 30 cycles of 95°C for 10 seconds, 50°C for 30 seconds, 72°C for 30 seconds. TaqMan PCR was run at 95°C for 5 minutes, then 40 cycles of 95°C for 10 seconds, 50°C for 40 seconds. Control, parallel tubes for PCR were kept at room temperature for 90 minutes. Flocculent precipitations were observed when ratio of blood was more than 1% in PCR and TaqMan PCR reaction, but not the LF-RPA.
(PDF)
