# Supplementary figures and images for: Rapid detection of Burkholderia pseudomallei with a lateral flow recombinase polymerase amplification assay
Source: PLoS One. 2019 Jul 8;14(7):e0213416. doi: 10.1371/journal.pone.0213416 (PMC6613700; doi:10.1371/journal.pone.0213416)

S2 Fig.

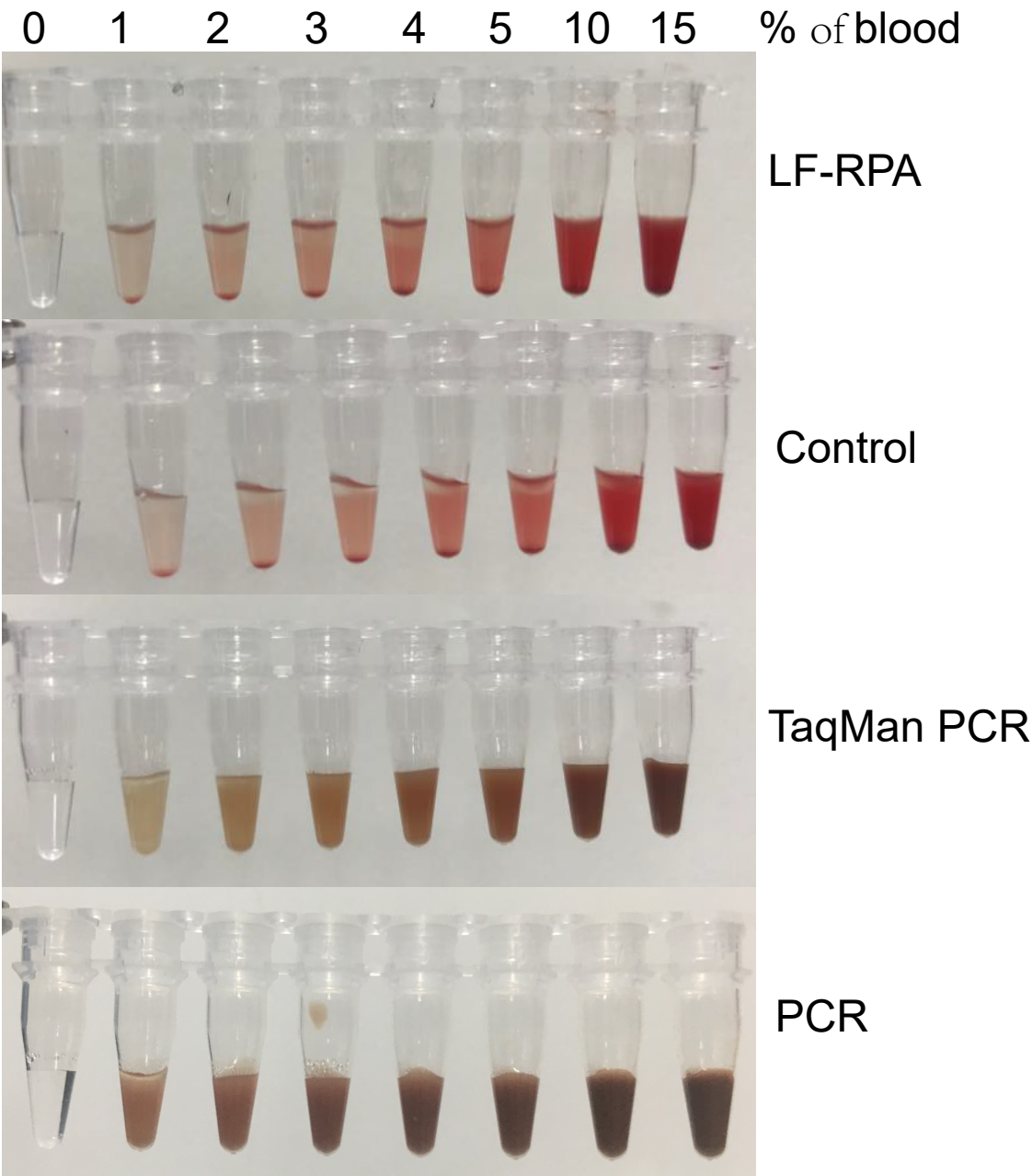

Supplement: S2 Fig — Defibrinated rabbit blood were proportionally added in the reactions of the LF-RPA, standard PCR or TaqMan PCR at the final concentration of 0%, 1%, 2% 3%, 4%, 5%,10% and 15% (v/v). The LF-RPA was conducted at 40°C for 20 minutes. PCR was performed at 95°C for 5minutes, then 30 cycles of 95°C for 10 seconds, 50°C for 30 seconds, 72°C for 30 seconds. TaqMan PCR was run at 95°C for 5 minutes, then 40 cycles of 95°C for 10 seconds, 50°C for 40 seconds. Control, parallel tubes for PCR were kept at room temperature for 90 minutes. Flocculent precipitations were observed when ratio of blood was more than 1% in PCR and TaqMan PCR reaction, but not the LF-RPA. (PDF) [file pone.0213416.s002.pdf]
